# Brown Algae-Derived Fucoidan Exerts Oxidative Stress-Dependent Antiproliferation on Oral Cancer Cells

**DOI:** 10.3390/antiox11050841

**Published:** 2022-04-26

**Authors:** Jun-Ping Shiau, Ya-Ting Chuang, Kun-Han Yang, Fang-Rong Chang, Jyh-Horng Sheu, Ming-Feng Hou, Jiiang-Huei Jeng, Jen-Yang Tang, Hsueh-Wei Chang

**Affiliations:** 1 Department of Surgery, Kaohsiung Medical University Hospital, Kaohsiung 80708, Taiwan; drshiaoclinic@gmail.com (J.-P.S.); mifeho@kmu.edu.tw (M.-F.H.); 2 Division of Breast Oncology and Surgery, Department of Surgery, Kaohsiung Medical University Chung-Ho Memorial Hospital, Kaohsiung Medical University, Kaohsiung 80708, Taiwan; 3 Department of Biomedical Science and Environmental Biology, College of Life Science, Kaohsiung Medical University, Kaohsiung 80708, Taiwan; u107023007@gap.kmu.edu.tw; 4 Graduate Institute of Natural Products, Kaohsiung Medical University, Kaohsiung 80708, Taiwan; kunhan1013@gap.kmu.edu.tw (K.-H.Y.); aaronfrc@kmu.edu.tw (F.-R.C.); 5 Department of Marine Biotechnology and Resources, National Sun Yat-sen University, Kaohsiung 80424, Taiwan; sheu@mail.nsysu.edu.tw; 6 Department of Medical Research, China Medical University Hospital, China Medical University, Taichung 40402, Taiwan; 7 School of Dentistry, College of Dental Medicine, Kaohsiung Medical University, Kaohsiung 80708, Taiwan; jhjeng@kmu.edu.tw; 8 Department of Dentistry, Kaohsiung Medical University Hospital, Kaohsiung 80708, Taiwan; 9 Department of Dentistry, National Taiwan University Hospital, Taipei 100225, Taiwan; 10 School of Post-Baccalaureate Medicine, Kaohsiung Medical University, Kaohsiung 80708, Taiwan; 11 Department of Radiation Oncology, Kaohsiung Medical University Hospital, Kaoshiung Medical University, Kaohsiung 80708, Taiwan; 12 Center for Cancer Research, Kaohsiung Medical University, Kaohsiung 80708, Taiwan

**Keywords:** fucoidan, algae, dietary natural product, polysaccharide, oral cancer, oxidative stress

## Abstract

Fucoidan is a dietary brown algae-derived fucose-rich polysaccharide. However, the anticancer effects of fucoidan for oral cancer treatment remain unclear, particularly in terms of its preferential antiproliferation ability and oxidative-stress-associated responses. This study first evaluated the effects and mechanisms of the preferential antiproliferation of fucoidan between oral cancer and non-malignant oral cells (S–G). In a 48 h MTS assay, fucoidan showed higher antiproliferation in response to five types of oral cancer cells, but not S–G cells, demonstrating preferential antiproliferation of oral cancer cells. Oral cancer cells (Ca9-22 and CAL 27) showing high sensitivity to fucoidan were selected to explore the antiproliferation mechanism compared to S–G cells. Fucoidan showed subG1 accumulation and an annexin V increase in apoptosis, accompanied by caspase 8, 9, and 3 activations in oral cancer cells, but not in S–G cells. Fucoidan increased reactive oxygen species and mitochondrial superoxide levels and decreased cellular glutathione in oral cancer cells compared with S–G cells. These oxidative stress effects were attributed to the downregulation of antioxidant signaling genes (*NRF2*, *TXN*, and *HMOX1*) in oral cancer cells rather than S–G cells. Fucoidan showed DNA damage-inducible effects (γH2AX and 8-hydroxy-2-deoxyguanosine) in oral cancer cells but not in S–G cells. Accordingly, these preferential changes in oral cancer but not in non-malignant cells contribute to the preferential antiproliferation mechanism of fucoidan. Furthermore, these changes were reverted by pretreatment with the antioxidant *N*-acetylcysteine. Therefore, for the first time, this study provides a detailed understanding of the preferential antiproliferation effects and mechanisms of fucoidan in oral cancer cells.

## 1. Introduction

Oral cancer is the most common subtype of head and neck malignancy, with a high disease burden [1]. Besides surgical eradication, chemotherapy and radiotherapy are helpful adjuvant therapies in oral cancer treatment, albeit at the cost of severe adverse events [2]. To avoid side effects, preferential antiproliferation agents, which exhibit antiproliferation in cancer cells but spare normal cells, are highly promising candidates as anticancer drugs. Thus, developing natural products with preferential antiproliferation effects is a sensible strategy for anti-oral cancer therapy.

Dietary natural products are commonly non-toxic. Marine algae are popular dietary foods as nutritional and functional resources with non-cytotoxicity [3]. Marine algae are rich in bioactive polysaccharides [4,5]. Sulfated polysaccharides, such as fucoidans, carrageenans, and ulvans, are abundant in brown, red, and green algae [4]. For example, fucoidan is a fucose-rich polysaccharide extracted from brown algae such as *Fucus vesiculosus*, *Fucus evanescens*, *Alaria esculenta*, *Ascophyllum nodosum*, *Macrocystis pyrifera*, *Sargassum polycystum*, and *Laminaria japonicia* [6].

Fucoidan exhibits anti-inflammatory [7], anticoagulant [8], antibacterial [9], and anticancer [10] effects. Its antitumor and apoptosis effects are regulated by mitogen-activated protein kinase (MAPK) through the upregulation of extracellular signal-regulated kinase (ERK1/2) and the downregulation of p38 MAPK and protein kinase B (AKT) [11,12,13]. Moreover, in vivo studies demonstrated that fucoidan inhibits breast tumor growth [14]. Fucoidan was certified as a safe food ingredient by the United States Food and Drug Administration (FDA) in the GRAS category [15]. Accordingly, fucoidan is a potential anticancer agent without side effects.

Fucoidan-induced apoptosis is validated to depend on oxidative stress in the example of bladder cancer cells [16]. Besides apoptosis, oxidative stress also modulates cellular changes such as DNA damage [17] and antioxidant signaling [18,19,20]. However, the antiproliferation mechanisms exerted by fucoidan involving oxidative stress-associated responses to cancer cells have not been thoroughly investigated. In particular, oxidative stress, DNA damage, apoptosis, and antioxidant signaling in fucoidan-treated oral cancer cells have rarely been examined.

This investigation focused on evaluating the preferential antiproliferation impact of fucoidan against oral cancer cells but not normal cells. The detailed mechanism of fucoidan-exposed oral cancer cells was explored, particularly for preferential apoptosis, oxidative stress, antioxidant signaling, and DNA damage.

## 2. Materials and Methods

### 2.1. Chemicals

Fucoidan derived from *Fucus vesiculosus* was obtained from Carbosynth (Compton, Berkshire, UK). Oxidative stress inhibitor *N*-acetylcysteine (NAC) [21,22] (Sigma-Aldrich; St. Louis, MO, USA) was used for pretreatments (10 mM, 1 h) and co-treatment (10 mM for 36 or 48 h) as indicated in the figure legends. Both fucoidan and NAC were dissolved in 1× PBS buffer before experiments.

### 2.2. Cell Cultures

CAL 27, HSC-3 (ATCC; Manassas, VA, USA), and Ca9-22 (RIKEN BioResource Research Center; Tsukuba, Ibaraki, Japan) oral cancer cell lines were included. The OC-2 [23] and OECM-1 [24] oral cancer cell lines were provided by Dr. Wan-Chi Tsai (Kaohsiung Medical University, Kaohsiung, Taiwan). They were established from gingival (Ca9-22 and OCEM-1), tongue (CAL 27 and HSC-3), and buccal mucosa (OC-2) oral squamous cell carcinoma (OSCC). A non-malignant normal gingival epithelial Smulow–Glickman (S–G) cell line [25,26], commonly used for examining the safety of anti-oral cancer drugs [27], was chosen as the control. Cells were maintained in a 3:2 medium mixture of DMEM and F12 (Gibco, Grand Island, NY, USA), supplemented with 10% fetal bovine serum and P/S antibiotics [28]. For all flow cytometry experiments, cells were seeded at a density of 4 × 10^4^/well/12-well plate and incubated overnight before drug treatment.

### 2.3. Cell Viability Assays

A tetrazolium-based cell viability detecting MTS kit (Promega Corporation, Madison, WI, USA), was applied. Ca9-22, CAL 27, and S–G cells were seeded at a density of 4, 4, and 6 × 10^3^/well/96-well plate for 48 h viability assays, and 2, 2, and 3 × 10^3^/well/96-well plate for 72 h viability assays, respectively. The cells were then incubated overnight before drug treatment. After drug treatment, cells were reacted with an MTS reagent for 1 h. Cell viability is proportional to colored formazan dye and measured by an ELISA reader at 490 nm [29].

### 2.4. Cell Cycle Assays

Cells were fixed and then mixed with 7-aminoactinmycin D (7AAD) (1 μg/mL) (Biotium; Hayward, CA, USA) for a 30 min staining reaction [30]. DNA content is proportional to the 7AAD staining intensity, measured with a Guava easyCyte flow cytometer (Luminex, TX, USA) (FL3 channel). Finally, Flow Jo 10 software (Becton-Dickinson; Franklin Lakes, NJ, USA) was used for cell cycle determination.

### 2.5. Apoptosis (Annexin V/7AAD) Assays

Cells were double-stained with annexin V (1:1000) and 7AAD (1 μg/mL) [31] (Strong Biotech; Taipei, Taiwan) for 1 h at 37 °C. Apoptotic cells are proportional to the annexin V staining intensity, detected by a Guava easyCyte flow cytometer (FL1/FL3 channels). The annexin V (+) and 7AAD (+ or −) signals were assigned to apoptosis (+) cells.

### 2.6. Apoptosis (Caspases 3, 8, and 9) Assays

Caspase (Cas) 3, 8, and 9 activations are commonly examined to detect the involvements of the apoptosis executor and the extrinsic and intrinsic apoptosis signaling. Peptide-based kits for flow cytometry (OncoImmunin; Gaithersburg, MD, USA) were applied to detect Cas 3, 8, and 9 [32,33]. Cells were supplemented with a 10 μM peptide mixture (1:1000) and stood at 37 °C for 1 h. Cleavage of substrates such as PhiPhiLux-G1D2, CaspaLux8-L1D2, and CaspaLux9-M1D2 by the activated Cas 3, 8, and 9, respectively, produced green fluorescence for flow cytometry analysis (FL1 channel). The actual setting for the windows is presented in Supplementary Figure S1.

### 2.7. Reactive Oxygen Species (ROS), Mitochondrial Superoxide (MitoSOX), and Glutathione (GSH) Assays

ROS production, MitoSOX generation, and GSH depletion were used to examine oxidative stress changes, which were detected by 2′,7′-dichlorodihydrofluorescein diacetate (H_2_DCFDA) (Sigma-Aldrich, St. Louis, MO, USA) (10 μM, 30 min) [28], MitoSOX™ Red (50 nM, 30 min), and 5-chloromethylfluorescein diacetate (CMF-DA) (Thermo Fisher Scientific, Carlsbad, CA, USA) (5 μM, 20 min) [29], for ROS, MitoSOX, and GSH, respectively. After the reaction in darkness at 37 °C, these detecting dyes became fluorescent and were conducive to flow cytometry analysis (FL1, FL2, and FL1 channels for ROS, MitoSOX, and GSH, respectively). The actual setting for the windows is presented in Supplementary Figure S2.

### 2.8. Quantitative PCR (qPCR)

RNA was extracted for cDNA conversion by Trizol solvent (Invitrogen, San Diego, CA, USA) [34] and an OmniScript RT kit (Qiagen, Valencia, CA, USA) [35]. Antioxidant signaling and housekeeper genes, including NFE2-like BZIP transcription factor 2 (*NRF2*; *NFE2L2*), thioredoxin (*TXN*), and heme oxygenase 1 (*HMOX1*) (Table 1), were included for qPCR under the touch-down program [35]. The mRNA expression level was assessed by 2^−ΔΔCt^ criteria compared with the glyceraldehyde-3-phosphate dehydrogenase (*GAPDH*) gene.

### 2.9. γH2AX/7AAD and 8-Hydroxy-2-Deoxyguanosine (8-OHdG) Detections

DNA damage markers γH2AX and 8-OHdG [36] were examined. For γH2AX measurement, cells were fixed and mixed with an antibody for γH2AX [36] (Santa Cruz Biotechnology; Santa Cruz, CA, USA) (4 °C, 1 h) and Alexa Fluor 488 secondary antibody (Cell Signaling Technology, Danvers, MA, USA). Then, cells were mixed with 7AAD (5 μg/mL, 30 min). In terms of 8-OHdG detection, cells were fixed and incubated with an FITC-8-OHdG antibody (Santa Cruz Biotechnology, Santa Cruz, CA, USA) (4 °C, 1 h). Finally, they were analyzed by flow cytometry (FL1/FL3 and FL1 channels for γH2AX/7AAD and 8-OHdG, respectively). The actual settings for the windows are presented in Supplementary Figures S3 and S4.

### 2.10. Statistical Analysis

ANOVA and a post hoc test (JMP 14 software; SAS Institute Inc., Cary, NC, USA) were chosen for statistical evaluation. The post hoc connecting letters are shown in each treatment. Different connecting lowercase letters represent significant differences for multi-comparison. Examples are provided in each figure legend to elucidate the results.

## 3. Results

### 3.1. Preferential Antiproliferation Effect of Fucoidan

**Figure 1:**
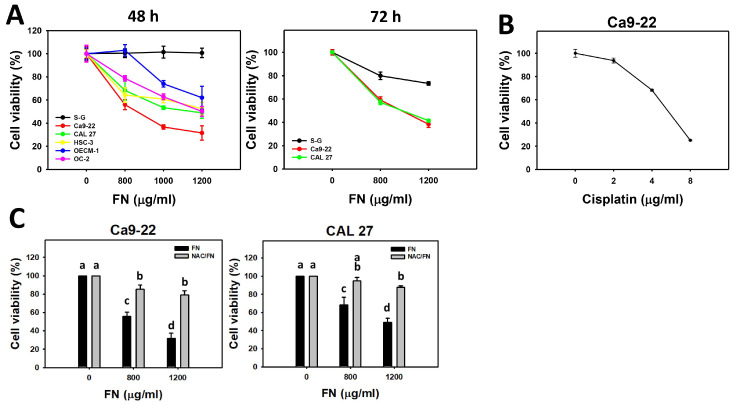
Fucoidan (FN) decreases the cell viability of oral cancer cells but not non-malignant cells. (**A**) MTS assay. After exposure to fucoidan (control, 800, 1000, and 1200 μg/mL) for 48 and 72 h, an MTS kit was chosen to determine the cell viability of oral cancer (Ca9-22, CAL 27, OECM-1, HSC-3, and OC-2) and non-malignant oral cells (S–G). (**B**) Cisplatin was a positive control for oral cancer cells (Ca9-22) following 24 h treatment. (**C**) The effect of oxidative stress on the cell viability of oral cancer cells following fucoidan treatment. After NAC (10 mM, 1 h) preincubation or not, cells were post-treated with control and fucoidan (0, 800, and 1200 μg/mL) with NAC co-treatment (10 mM) for 48 h. NAC/fucoidan (NAC/FN) represents pretreatment with NAC and post-treatment with FN. Data = mean ± SD (*n* = 3 experiments). Data of the same cells with non-overlapping letters reveal significant differences (*p* < 0.05). In the example of Figure 1B (Ca9-22 cells), FN at 0, 800, and 1200 μg/mL labeled with “a, c, and d” has significant results because the letters do not overlap. FN and NAC/FN of Ca9-22 cells at 800 or 1200 μg/mL labeled with “c and b” or “d and b” have significant results.

The 48 h cell viability (%) was dose-dependently decreased by fucoidan in oral cancer cells (Ca9-22, CAL 27, OC-2, HSC-3, and OECM-1) but was maintained in non-malignant oral cells (S–G) (Figure 1A). Similarly, the 72 h cell viability (%) was dose-dependently decreased by fucoidan in oral cancer cells (Ca9-22 and CAL 27), and S–G cells still showed higher viability than oral cancer cells. Hence, fucoidan-treated oral cancer cells exhibited preferential antiproliferation, but non-malignant oral cells did not. Due to their high sensitivity to fucoidan, the oral cancer cells (Ca9-22 and CAL 27) were used to explore the detailed mechanisms in the following experiments. Cisplatin was a positive control for oral cancer cells (Ca9-22) following 24 h treatment (Figure 1B). Moreover, antioxidant NAC reversed the fucoidan-induced antiproliferation in oral cancer cells (Figure 1C), suggesting that oxidative stress was involved.

### 3.2. Cell Cycle Effect of Fucoidan

**Figure 2:**
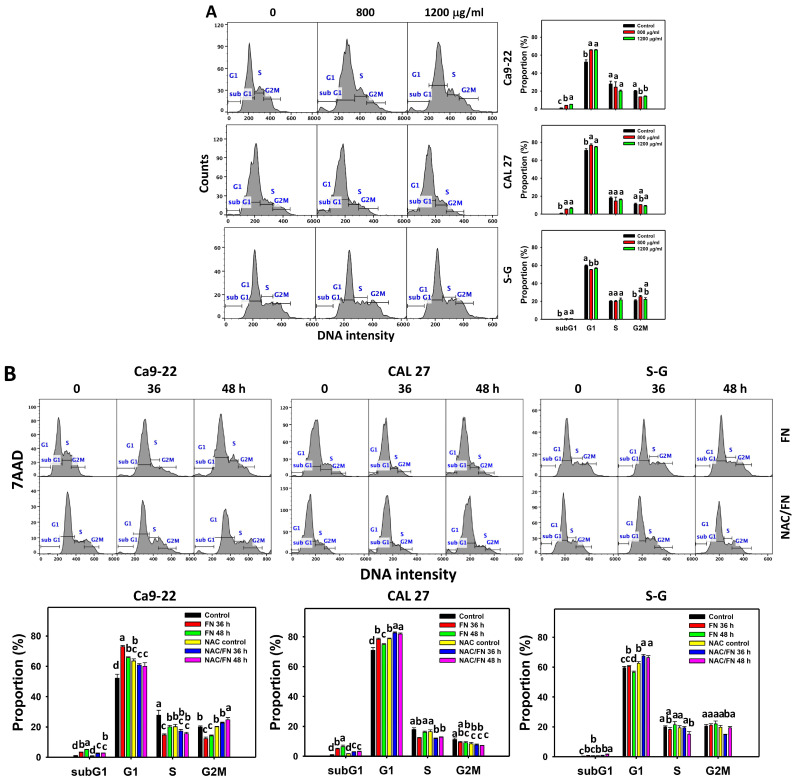
Fucoidan increases subG1 populations of oral cancer cells but not non-malignant cells. (**A**) Cell cycle distribution and statistical analysis. Oral cancer (Ca9-22 and CAL 27) and non-malignant oral cells (S–G) were treated with fucoidan (control, 800 (FN 800), and 1200 (FN 1200) μg/mL) for 0 and 48 h. (**B**) The effect of oxidative stress on the cell cycle change of fucoidan-exposed oral cancer cells. After NAC (10 mM, 1 h) preincubation or not, cells were treated with fucoidan (1200 μg/mL) with NAC co-treatment (10 mM) for 0, 36, and 48 h. NAC/fucoidan (NAC/FN) represents pretreatment with NAC and post-treatment with FN. Data = mean ± SD (*n* = 3 experiments). Data with non-overlapping letters reveal significant differences for each phase (*p* < 0.05). In the example of Figure 2A (subG1 phase of Ca9-22 cells), FN at 0, 800, and 1200 μg/mL labeled with “d, b, and a” has significant results because the letters do not overlap. In the example of Figure 2B (G1 phase of Ca9-22 cells), control, FN 36 h, and FN 48 h labeled with “d, a, and b” have significant results because the letters do not overlap. In the example of Figure 2B (G2/M phase of Ca9-22 cells), FN 48 h and NAC/FN 48 h labeled with “c and a” have significant results. The positive control of G2/M arrest of the cell cycle is provided in Supplementary Figure S5.

After fucoidan incubation, the cell cycle histograms in oral cancer and non-malignant oral cells (S–G) were generated (Figure 2A). For dose and time experiments (Figure 2A,B), fucoidan-incubated oral cancer cells (Ca9-22 and CAL 27) showed higher subG1 and G1 populations and lower G2/M populations than the controls. In contrast, fucoidan-incubated S–G cells showed no subG1 accumulation (Figure 2A,B). Moreover, in the dose experiment, fucoidan-incubated S–G cells showed lower G1 at 800 and 1200 μg/mL and higher G2/M populations at 800 μg/mL, while they showed no G2/M changes at 1200 μg/mL. In the time experiments, fucoidan-incubated S–G cells showed no change of cell cycle phases compared to the controls except for lower G1 at 48 h (Figure 2B).

Furthermore, the impact of oxidative stress on cell cycle progression was concerning, due to the NAC effect on rescuing viability in fucoidan-treated oral cancer cells (Figure 1B). The antioxidant NAC reversed the fucoidan-induced subG1 accumulation in oral cancer cells (Figure 2B). Moreover, NAC decreased the fucoidan-induced G1 increment and increased G2/M phases of oral cancer cells (Ca9-22). In contrast, NAC increased the fucoidan-induced G1 increment and decreased G2/M phases of oral cancer (CAL 27) and S–G cells. These results suggest that oxidative stress is essential to fucoidan-regulated cell cycle progression.

### 3.3. Preferential Apoptosis Effect of Fucoidan

**Figure 3:**
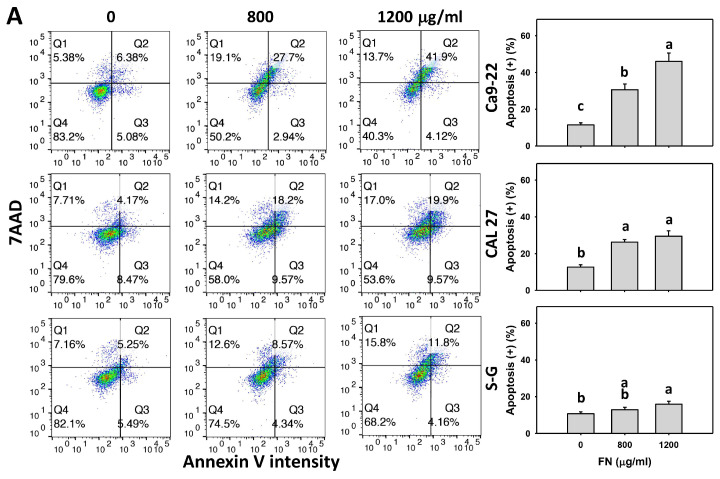

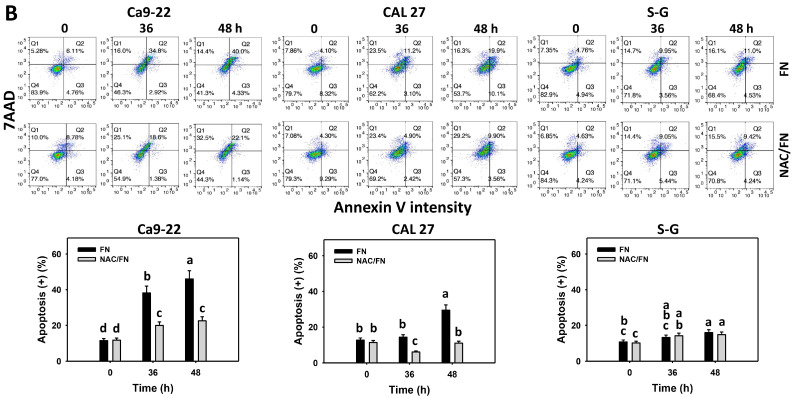
Fucoidan drives apoptosis (annexin V) in oral cancer cells. (**A**) Annexin V histogram and statistical analysis. Oral cancer (Ca9-22 and CAL 27) and non-malignant cells (S–G) were treated with fucoidan (control, 800, and 1200 μg/mL) for 0 and 48 h. Annexin V (+) and 7AAD (+ or −) counts, i.e., Q2 and Q3 quadrants, were regarded as apoptosis (%). It is noted that some of the Q2 cells could be necrotic and not apoptotic. Q4 and Q1 quadrants were regarded as live cells and necrosis (%). For the Q1 quadrant, the DNA staining (7AAD) indicates that the cell membrane has been permeabilized, but Annexin V does not enter and stain the cells; perhaps it is too big (35 kD), whereas 7AAD (1.27 kD) is small enough to enter. (**B**) The effect of oxidative stress on apoptosis (annexin V) of fucoidan-incubated oral cancer cells. After NAC preincubation or not, cells were treated with fucoidan (1200 μg/mL) with NAC co-treatment (10 mM) for 0, 36, and 48 h. In general, the actual setting for the windows in (**A**) is based on a similar annexin V intensity (+) (%) to the controls between different cell lines. For NAC pretreatment, the actual setting for the windows in (**B**) is based on the same annexin V intensity of the controls between different cell lines. NAC/fucoidan (NAC/FN) represents the pretreatment with NAC and post-treatment with FN. Data = mean ± SD (*n* = 3 experiments). Data with non-overlapping letters reveal significant differences (*p* < 0.05). In the example of Figure 3B (Ca9-22 cells), FN at 0, 36, and 48 h, labeled with the connecting letters “d, b, and a”, has significant results, because the letters do not overlap. FN and NAC/FN at 48 h labeled with “a and c” have significant results. In contrast, in the example of Figure 3B (S–G cells), FN and NAC/FN at 36 h labeled with “abc and ab” have nonsignificant results because they overlap with “ab”. The positive control of apoptosis is provided in Supplementary Figure S6.

Annexin V detects phosphatidylserine flipflopping to the outer plasma membrane, proportional to apoptosis. Based on the dose and time experiments for annexin V/7ADD analysis, fucoidan increased the annexin V (+) populations in oral cancer cells (Ca9-22 and CAL 27) more than in non-malignant oral cells (S–G) (Figure 3A,B), indicating that fucoidan causes preferential apoptosis in oral cancer cells.

Furthermore, the impact of oxidative stress in regulating apoptosis was of concern. The antioxidant NAC reversed the fucoidan-induced annexin V increment in oral cancer cells (Figure 3B), suggesting that oxidative stress is essential to fucoidan-induced apoptosis.

### 3.4. Preferential Apoptosis Signaling Effect of Fucoidan

**Figure 4:**
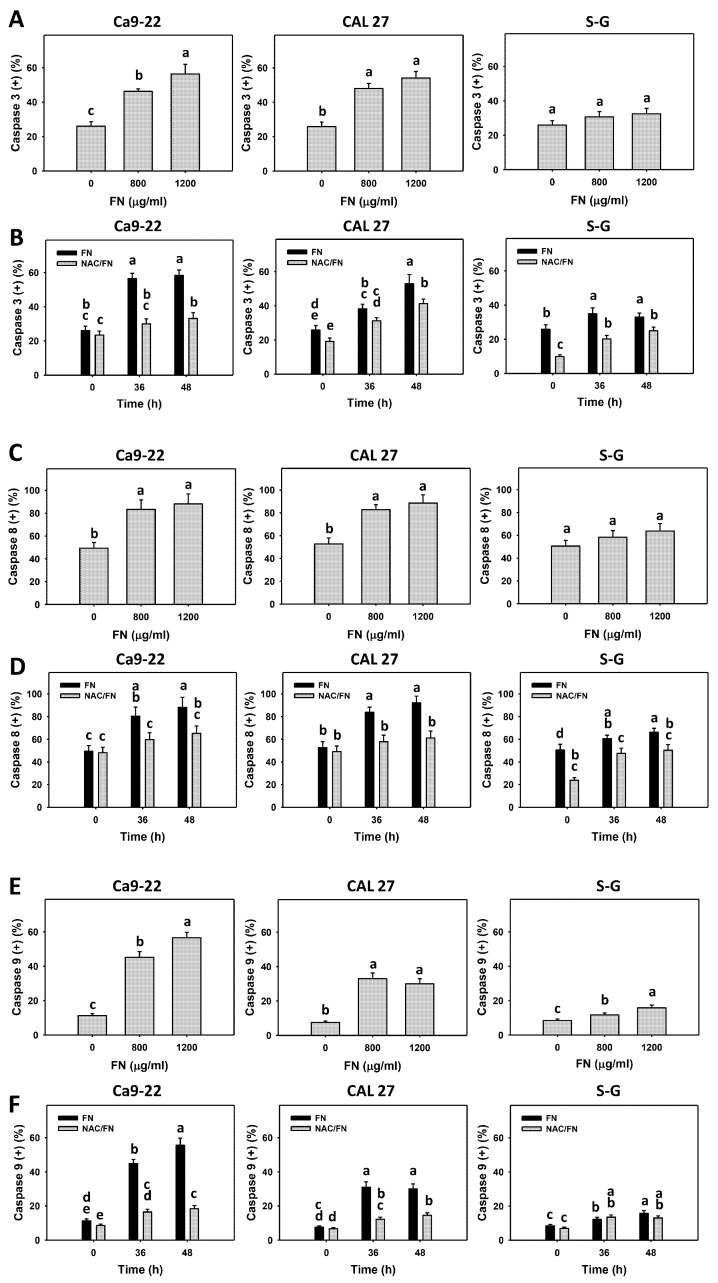
Fucoidan drives Cas 3, 8, and 9 signaling in oral cancer cells. Summary histograms of the flow cytometry results are presented. The individual density plots are presented in Supplementary Figure S1. (**A**,**C**,**E**) Cas 3, 8, and 9 statistical analysis. Oral cancer (Ca9-22 and CAL 27) and non-malignant cells (S–G) were treated with fucoidan (control, 800, and 1200 μg/mL) for 0 and 48 h. (+) indicates a high level of Cas 3, 8, and 9. (**B**,**D**,**F**) The effect of oxidative stress on Cas 3, 8, and 9 activations of fucoidan-incubated oral cancer cells. After NAC preincubation or not, cells were treated with fucoidan (1200 μg/mL) with NAC co-treatment (10 mM) for 0, 36, and 48 h. NAC/fucoidan (NAC/FN) represents the pretreatment with NAC and post-treatment with FN. Data = mean ± SD (*n* = 3 experiments). Data with non-overlapping letters reveal significant differences (*p* < 0.05). In the example of Figure 4B (Ca9-22 cells), FN at 36 and 48 h labeled with “a” has significant results compared with that at 0 h labeled with “bc”, because the letters do not overlap. FN and NAC/FN at 36 and 48 h labeled with “a and bc” and “a and b” have significant results. In contrast, FN at 36 and 48 h labeled with “a” has nonsignificant results because the letters overlap. e is not appeared for statistic analysis in the example.

Cas 3, 8, and 9 activation is detected by peptide cleavage to generate fluorescence, proportional to the activation of these apoptosis signals as detected by flow cytometry. Flow cytometry density plots are presented in Supplementary Figure S1. Based on the dose (Figure 4A,C,E) and time (Figure 4B,D,F) experiments, fucoidan increased Cas 3, 8, and 9 (+) populations more in oral cancer cells (Ca9-22 and CAL 27) than in non-malignant oral cells (S–G), indicating that fucoidan causes the preferential activation of Cas 3, 8, and 9 in oral cancer cells.

Furthermore, the impact of oxidative stress on Cas 3, 8, and 9 activations was of concern. The antioxidant NAC reversed the fucoidan-induced Cas 3, 8, and 9 increments in oral cancer cells (Figure 4B,D,F), suggesting that oxidative stress is essential to fucoidan-induced Cas 3, 8, and 9 activations.

### 3.5. Preferential ROS and MitoSOX Generations, and GSH Depletion Effects of Fucoidan

**Figure 5:**
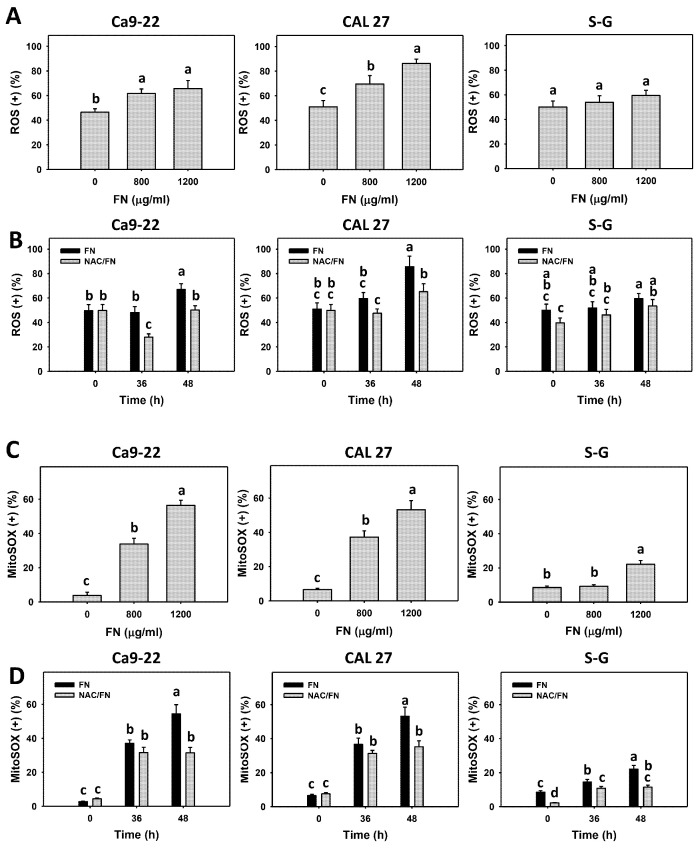
Fucoidan drives ROS and MitoSOX generation in oral cancer cells. Summary histograms of the flow cytometry results are presented. The individual density plots are presented in Supplementary Figure S2. (**A**,**C**) ROS and MitoSOX statistical analyses. Oral cancer (Ca9-22 and CAL 27) and non-malignant cells (S–G) were treated with fucoidan (control, 800, and 1200 μg/mL) for 0 and 48 h. (+) indicates high levels of ROS and MitoSOX. (**B**,**D**) The effect of oxidative stress on ROS and MitoSOX generation and the GSH depletion of fucoidan-incubated oral cancer cells. After NAC preincubation or not, cells were treated with fucoidan (1200 μg/mL) with NAC co-treatment (10 mM) for 0, 36, and 48 h. NAC/fucoidan (NAC/FN) represents the pretreatment with NAC and post-treatment with FN. Data = mean ± SD (*n* = 3 experiments). Data with non-overlapping letters reveal significant differences (*p* < 0.05). In the example of Figure 5D (Ca9-22 cells), FN at 0, 36, and 48 h, labeled with the connecting letters “c, b, and a”, has significant results, because the letters do not overlap. FN and NAC/FN at 48 h labeled with “a and b” have significant results. In contrast, FN and NAC/FN at 36 h labeled with “b” have nonsignificant results because they overlap with “b”. “d” is not appeared for statistic analysis in the example. The positive controls of ROS and MitoSOX are provided in Supplementary Figures S7 and S8.

**Figure 6:**
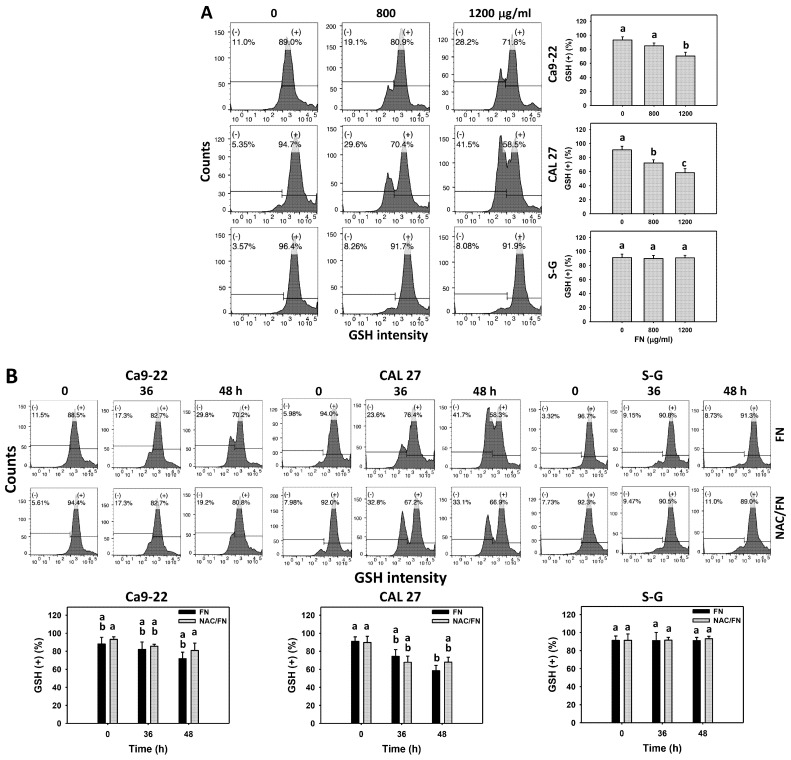
Fucoidan drives GSH depletion in oral cancer cells. (**A**) GSH flow cytometry density plots and summary histogram analyses. Oral cancer (Ca9-22 and CAL 27) and non-malignant cells (S–G) were treated with fucoidan (control, 800, and 1200 μg/mL) for 0 and 48 h. (+) indicates high levels of GSH. (**B**) The effect of oxidative stress on the GSH depletion of fucoidan-incubated oral cancer cells. After NAC preincubation or not, cells were treated with fucoidan (1200 μg/mL) with NAC co-treatment (10 mM) for 0, 36, and 48 h. In general, the actual setting for the windows is based on the same GSH intensity of the controls between different cell lines. NAC/fucoidan (NAC/FN) represents the pretreatment with NAC and post-treatment with FN. Data = mean ± SD (*n* = 3 experiments). Data with non-overlapping letters reveal significant differences (*p* < 0.05). In the example of Figure 6A (CAL 27 cells), FN at 0, 800, and 1200 μg/mL, labeled with the connecting letters “a, b, and c”, has significant results, because the letters do not overlap. In contrast, in the example of Figure 6A (S–G cells), FN at 0, 800, and 1200 μg/mL labeled with “a” has nonsignificant results because they overlap with “a”.

Oxidative stress (ROS and MitoSOX) and the cellular antioxidant GSH were detected. Based on the dose (Figure 5A,C) and time (Figure 5B,D) experiments, fucoidan increased ROS and MitoSOX (+) populations more in oral cancer cells (Ca9-22 and CAL 27) than in non-malignant oral cells (S–G). Moreover, fucoidan decreased GSH (+) populations more in oral cancer cells than in S–G cells (Figure 6A,B). After fucoidan treatment, the GSH level remained unchanged in S–G cells. These results indicate that fucoidan causes preferential oxidative stress and GSH depletion in oral cancer cells.

Furthermore, the impact of oxidative stress and GSH changes was of concern. The antioxidant NAC reversed the fucoidan-induced ROS and MitoSOX increments (Figure 5B,D). NAC partly reversed the GSH depletion at 48 h treatment in oral cancer cells (Figure 6B), which indicates that oxidative stress is essential for fucoidan-induced ROS and MitoSOX generation, accompanied by GSH depletion.

### 3.6. Preferential Antioxidant Signaling Effects of Fucoidan

**Figure 7:**
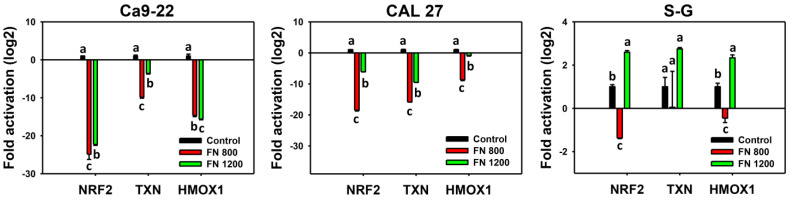
Fucoidan stimulates antioxidant signaling in oral cancer cells. Oral cancer (Ca9-22 and CAL 27) and non-malignant cells (S–G) were treated with fucoidan (control, 800 (FN 800), and 1200 (FN 1200) μg/mL) for 0 and 24 h for qPCR analysis. Data = mean ± SD (*n* = 3 experiments). Data with non-overlapping letters reveal significant differences (*p* < 0.05). In the example of Ca9-22 cells (HOMX1 gene), control, FN 800, and FN 1200, labeled with the connecting letters “a, b, and c”, have significant results, because the letters do not overlap.

The inhibition of antioxidant signaling leads to oxidative stress generation [37]. After fucoidan incubation (0, 800, and 1200 μg/mL), the mRNA expression of antioxidant signaling genes, such as *NRF2*, *TXN*, and *HMOX1*, was detected. Fucoidan dramatically downregulated these antioxidant genes in oral cancer cells (Ca9-22 and CAL 27) but not in non-malignant oral cells (S–G) (Figure 7). These antioxidant genes were maintained at a basal level at 800 μg/mL in S–G cells and slightly upregulated at 1200 μg/mL. These results indicate that fucoidan causes the preferential suppression of antioxidant signaling in oral cancer cells.

### 3.7. Preferential DNA Damage Effect of Fucoidan

**Figure 8:**
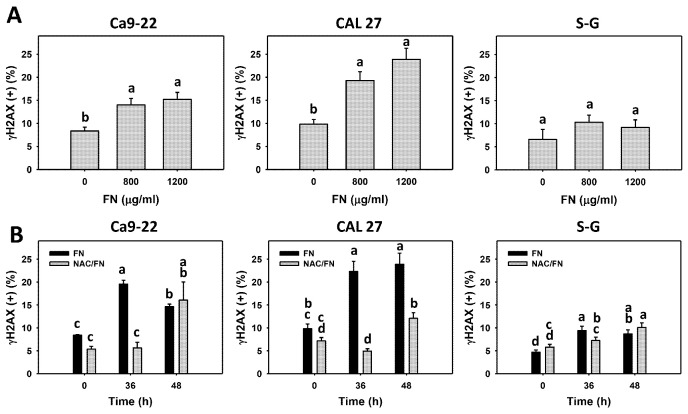
Fucoidan increases the γH2AX level of oral cancer cells. Summary histograms of the flow cytometry results are presented. The individual density plots are presented in Supplementary Figure S3. (**A**) γH2AX statistical analysis. Oral cancer (Ca9-22 and CAL 27) and non-malignant cells (S–G) were treated with fucoidan (control, 800, and 1200 μg/mL) for 0 and 48 h. (+) indicates a high level of γH2AX. (**B**) The effect of oxidative stress on the γH2AX increment in fucoidan-incubated oral cancer cells. After NAC preincubation or not, cells were post-treated with fucoidan (1200 μg/mL) with NAC co-treatment (10 mM) for 0, 36, and 48 h. NAC/fucoidan (NAC/FN) represents pretreatment with NAC and post-treatment with FN. Data = mean ± SD (*n* = 3 experiments). Data with non-overlapping letters reveal significant differences (*p* < 0.05). In the example of Figure 8B (CAL 27 cells), FN at 0 and 36 h, labeled with the connecting letters “bc and a”, has significant results, because the letters do not overlap. FN and NAC/FN at 36 or 48 h labeled with “a and d” and “a and b” have significant results. In contrast, FN at 36 and 48 h labeled with “a” has nonsignificant results because they overlap with “a”. The positive control of γH2AX is provided in Supplementary Figure S9.

**Figure 9:**
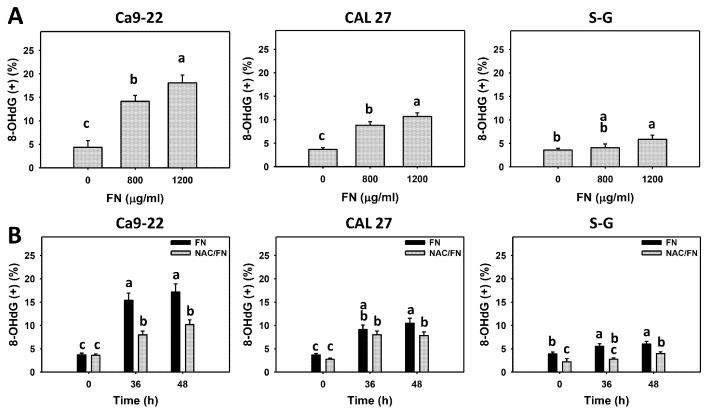
Fucoidan increases the 8-OHdG level of oral cancer cells. Summary histograms of the flow cytometry results are presented. The individual density plots are presented in Supplementary Figure S4. (**A**) 8-OHdG statistical analysis. Oral cancer (Ca9-22 and CAL 27) and non-malignant cells (S–G) were treated with fucoidan (control, 800, and 1200 μg/mL) for 0 and 48 h. (+) indicates a high level of 8-OHdG. (**B**) The effect of oxidative stress on the 8-OHdG increment in fucoidan-incubated oral cancer cells. After NAC preincubation or not, cells were treated with fucoidan (1200 μg/mL) with NAC co-treatment (10 mM) for 0, 36, and 48 h. NAC/fucoidan (NAC/FN) represents the pretreatment with NAC and post-treatment with FN. Data = mean ± SD (*n* = 3 experiments). Data with non-overlapping letters reveal significant differences (*p* < 0.05). In the example of Figure 9B (Ca9-22 cells), FN at 0 and 36 h, labeled with the connecting letters “c and a”, has significant results, because the letters do not overlap. FN and NAC/FN at 36 or 48 h labeled with “a and b” have significant results. In contrast, FN at 36 and 48 h labeled with “a” has nonsignificant results because they overlap with “a”. The positive control of 8-OHdG is provided in Supplementary Figure S10.

DNA damage, such as γH2AX and 8-OHdG, was detected. Fucoidan increased the populations of γH2AX (+) and 8-OHdG (+) in oral cancer cells (Ca9-22 and CAL 27) but not in non-malignant oral cells (S–G) (Figure 8A and Figure 9A), indicating that fucoidan causes preferential DNA damage to oral cancer cells.

Furthermore, the impact of oxidative stress on DNA damage was of concern. The antioxidant NAC reversed the fucoidan-induced γH2AX and 8-OHdG increments in oral cancer cells (Figure 8B and Figure 9B), suggesting that oxidative stress is essential to fucoidan-induced γH2AX and 8-OHdG DNA damage.

## 4. Discussion

The anticancer functions of fucoidan in oral cancer cells have not been thoroughly investigated. How antioxidant signaling and the DNA damage response are associated with oxidative stress in fucoidan-treated oral cancer cells remains unclear. Moreover, most studies on the anticancer properties of fucoidan focused on the antiproliferation effects without considering their impacts on normal cells. The present investigation used both oral cancer and normal cells to confirm that fucoidan exhibited preferential antiproliferation of oral cancer cells without changing non-malignant oral cells. The detailed preferential antiproliferation mechanisms were discussed, as follows.

### 4.1. Fucoidan Induces Preferential Antiproliferation Effect

Fucoidan has been applied to several oral cancer studies. For example, fucoidan-based nanoparticles were developed to deliver the phosphoinositide 3-kinase α (PI3Kα) inhibitor BYL719 to head and neck squamous cell carcinoma (HNSCC) cells [38]. Fucoidan suppresses the invasion of oral cancer cells [39]. Fucoidan also exhibits anticancer effects against acute leukemia [11], lymphoma [11], and lung [40], breast [15], and bladder cancer [16]. However, their normal cell responses to fucoidan have not been evaluated. Their antiproliferation response to oral cancer cells has rarely been investigated.

Recently, antiproliferation studies were reported in oral cancer cells. For example, IC_50_ values of fucoidan for oral cancer cells (SCC15 and SCC25) were 218.2 and 508.5 μg/mL in a 24 h MTT assay [41,42]. Moreover, the cytotoxicity of normal cells has rarely been reported. For comparison, we found that the IC_50_ values of fucoidan for oral cancer cells (Ca9-22, CAL 27, HSC-3, and OC-2) were 856.1, 1096.9, 1193.9, and 1190.6 μg/mL, respectively, in a 48 h MTS assay, but the viability of non-malignant oral cells was unchanged. Accordingly, the present study found that fucoidan exhibited preferential antiproliferation in oral cancer cells but not in non-malignant oral cells.

In almost all tests in the present study, the gingival cancer Ca9-22 cells were more sensitive to fucoidan than the tongue cancer CAL 27 cells following fucoidan treatment. Similarly, dihydrosinularin [43] and sulfonyl chromen-4-ones (CHW09) [44] also show higher sensitivity to Ca9-22 cells than CAL 27 cells. In contrast, burmannic acid [33] and antimycin A [45] are more sensitive to CAL 27 cells than Ca9-22 cells. Therefore, the drug sensitivity of different oral cancer cell lines may depend on the nature of the drugs. The clinical drug cisplatin has IC_50_ values of 5.6 and 1.20 μg/mL in oral cancer Ca9-22 cells at the 24 h (Figure 1B) and 48 h MTS detection [46], respectively. Although oral cancer cells are more sensitive to cisplatin than fucoidan, cisplatin is frequently associated with adverse effects for clinical therapy [47].

### 4.2. Fucoidan Causes Preferential Oxidative Stress

Antioxidants show bifunctional effects for suppressing and inducing oxidative stress at physiologic and high concentrations, respectively [48]. Similarly, fucoidan is a potential radical scavenger [49] and protects against peroxide-induced damage in pre-osteoblastic cells [50]. Alternatively, fucoidan induces oxidative stress in breast (MCF-7) [51], bladder (5637) [16], acute myeloid leukemia (SKM-1) [52], colon (Caco-2) [53], liver (SMMC-7721) [54], and tongue (H103) [55] cancer cells. This is a slight paradox as the literature lists the strong antioxidative properties of fucoidan. Perhaps the antioxidative properties only apply to healthy cells. Accordingly, fucoidan may exhibit bifunctional effects, suppressing or inducing oxidative stress in different cell contexts to protect normal cells but damage cancer cells.

Oxidative stress is generated when cellular antioxidants, such as GSH, are downregulated. For example, anticancer drugs such as amygdalin exhibit oxidative stress in breast cancer cells by downregulating GSH levels [56]. Indoxyl sulfate induces GSH depletion, triggering oxidative stress in renal tubular cells [57]. Fucoidan causes oxidative stress, supported by the evidence of ROS and MitoSOX generation, as well as GSH depletion, in oral cancer cells (Figure 5A,C and Figure 6A). 

Furthermore, fucoidan stimulated more oxidative stress and induced more antioxidant depletion in oral cancer cells (Figure 5A,C and Figure 6A) than in non-malignant oral cells, demonstrating the preferential induction of oxidative stress in oral cancer cells by fucoidan. Cancer cells generally contain higher oxidative stress than non-malignant cells [58]. The preferential oxidative stress induction of fucoidan may exceed the oxidative stress threshold in oral cancer cells but is tolerated by non-malignant cells, causing preferential antiproliferation of oral cancer cells by fucoidan but not non-malignant cells.

### 4.3. Fucoidan Causes Preferential Downregulation of Antioxidant Signaling

Cellular redox homeostasis is maintained by balancing pro-oxidant and antioxidant levels [59,60]. NRF2, TXN, and GSH are essential members of cellular antioxidant signaling that diminish the excess of pro-oxidants [61,62]. The inhibition of antioxidant signaling may relatively enhance ROS levels [58].

Several natural products were reported to inhibit NRF2 signaling or directly suppress endogenous antioxidants, causing oxidative stress. For example, parthenolide induces ROS and downregulates NRF2 expression in breast cancer stem-like cells [63]. Diosmetin decreases NFR2 expression to enhance oxidative stress and apoptosis in lung cancer cells [64]. Moreover, TXN is a target of NRF2 [62]. NRF2 activates the TXN-associated antioxidant system [65] and also regulates HMOX1 [66]. Similarly, pomegranate extract (POMx) downregulates mRNA expressions of the *NRF2*, *TXN*, and *HMOX1* genes to induce oxidative stress in oral cancer Ca9-22 cells [67]. Accordingly, the antioxidant NRF2–TXN–HMOX1 axis is responsible for regulating cellular oxidative stress.

According to this rationale, we examined the antioxidant signaling by mRNA expressions in fucoidan-exposed oral cancer and non-malignant cells. Antioxidant genes (*NRF2*, *TXN*, and *HMOX1*) were downregulated in fucoidan-treated oral cancer cells but not in non-malignant oral cells (Figure 7). Fucoidan seems to downregulate the antioxidant signaling in oral cancer cells and subsequently stimulate oxidative stress by fucoidan, but not in non-malignant oral cells, causing preferential downregulation of antioxidant signaling in oral cancer cells, which may contribute to the preferential upregulation of oxidative stress and lead to preferential antiproliferation.

### 4.4. Fucoidan Causes Preferential Apoptosis

Oxidative stress modulation is a common strategy to induce apoptosis for cancer treatment [58,68]. Fucoidan triggers apoptosis in breast [51], bladder [16], leukemia [52], colon [53], and liver cancer cells [54]. However, these studies have rarely investigated preferential apoptosis effects and mechanisms. In the present investigation, fucoidan increased subG1 oral cancer cell populations (Figure 2) and annexin V intensities (Figure 3). These apoptosis effects were verified to be associated with fucoidan-triggered apoptosis signals, such as Cas 3, 8, and 9 (Figure 4). Accordingly, fucoidan induces intrinsic and extrinsic apoptosis in oral cancer cells.

The preferential apoptosis may be partly attributed to preferential oxidative stress. For example, the mitochondrion-targeted lonidamine-peptide drug induces preferential killing of breast cancer cells but not normal cells, accompanied by inducing higher ROS levels in breast cancer cells than normal cells [69]. Similarly, apoptosis changes such as increases in annexin V and caspase signaling activation (Cas 3, 8, and 9) showed higher expressions in fucoidan-treated oral cancer cells than non-malignant oral cells, leading to preferential apoptosis in oral cancer cells and contributing to preferential antiproliferation.

### 4.5. Fucoidan Causes Preferential DNA Damage

Oxidative stress causes DNA damage, such as DNA double-strand breaks (γH2AX) and oxidative DNA damage (8-OHdG) [70,71]. Fucoidan generates DNA damage in several cancer cells, such as leukemia, breast [72], and colon [73] cancer cells. Some studies provided evidence of DNA damage using a comet assay [72,74] without investigating the detailed mechanisms. Another fucoidan study showed an increase in γH2AX in colon cancer cells [73], similar to the present study, as detected by flow cytometry (Figure 9).

Moreover, for the first time, we further found that fucoidan induced oxidative 8-OHdG DNA damage in oral cancer cells (Figure 9). Moreover, γH2AX and 8-OHdG DNA damages showed higher expressions in fucoidan-treated oral cancer cells than non-malignant oral cells, leading to preferential DNA damage in oral cancer cells, which may contribute to preferential antiproliferation. 

In addition to DNA damage, oxidative stress may act on other macromolecules such as lipids and proteins [70], causing lipid and protein peroxidation. A detailed examination of lipid and protein peroxidation for fucoidan in oral cancer cells is warranted in the future.

### 4.6. Fucoidan Causes Preferential Cell Cycle Arrest

Fucoidan arrests the cell cycle at G1 in colorectal cancer cells (HCT116) [73] and breast cancer cells (4T1) [14] and also causes arrest at G2 in oral cancer cells (SCC15). In the present investigation, fucoidan induced subG1 accumulation and a minor G1 increase in oral cancer cells (Ca9-22 and CAL 27), but it showed little changes in the cell cycle distribution in non-malignant oral cells. These results suggest that fucoidan exhibits different impacts on cell cycle progression for different cancer cells. Moreover, these subG1 and G1 increments were more remarkable in fucoidan-treated oral cancer cells than non-malignant oral cells, leading to preferential subG1 and G1 increments in oral cancer cells, which may contribute to preferential antiproliferation.

### 4.7. Preferential Oxidative Stress Plays a Vital Role in Fucoidan-Induced Preferential Antiproliferation Mechanisms

The several responses triggered by oxidative stress warrant a detailed investigation of the role of oxidative stress in fucoidan-treated oral cancer cells by using the oxidative stress inhibitor NAC. NAC has been reported to revert fucoidan-induced apoptosis and downregulations of telomerase reverse transcriptase (TERT), c-myc, Sp1 transcription factor, and AKT expressions in bladder cancer cells [16]. However, the effects of NAC reversion on fucoidan-induced changes have rarely been investigated in other cancer cells.

In the present investigation, we provided a different mechanism for fucoidan and validated the effects of NAC in oral cancer cells. NAC reversed fucoidan-induced preferential impacts on antiproliferation (Figure 1), subG1 accumulation (Figure 2), apoptosis (Figure 3 and Figure 4), oxidative stress (Figure 5 and Figure 6), and DNA damage (Figure 8 and Figure 9) in oral cancer cells but not in non-malignant oral cells. Therefore, fucoidan induces oxidative stress-mediated preferential antiproliferation mechanisms in oral cancer cells.

### 4.8. Limitation of Our Fucoidan-Treated Oral Cancer Cell Study

Fucoidan shows preferential antiproliferation in oral cancer cells. However, the in vivo role of fucoidan against oral cancer was not examined in the present study. A detailed future assessment of fucoidan using the orthotopic nude mouse model of oral cancer cells is warranted [75,76].

Therefore, the expected next step is to examine fucoidan in preclinical and clinical trials. As recently reviewed, clinical trials have been carried out in other cancer types [39,77]. The problem with clinical trials on fucoidan, according to Lin et al. [39], is its complex structure and different forms; oral absorption appears to be poor, and it cannot be accurately measured in the body. It is approved as a food supplement, but it has not been approved as a drug by the FDA. Luthuli et al. [77] reviewed anticancer effects against several cancer cells such as liver, breast, cervical, and melanoma cancer cells. However, these reviews did not discuss the role of fucoidan in oral cancer treatment [39,77]. 

Two clinical trials underway are on its safety and biodistribution [78,79], and there is also one on non-small cell lung cancer in which it is added to the patients’ normal chemotherapy [78]. The US Government Clinical Trial register lists a phase 2 study on squamous cell carcinomas of the head and neck that should be completed in 2023 [79]. It is necessary to continuously investigate more mechanisms of fucoidan activity to improve its future application in oral cancer treatment.

## 5. Conclusions

Fucoidan is a brown algae-derived fucose-rich polysaccharide. The anticancer effects of fucoidan on oral cancer cells have rarely been reported, especially with regard to its preferential antiproliferation ability and oxidative-stress-associated responses. The present investigation examined oxidative stress, DNA damage, apoptosis, and antioxidant signaling in fucoidan-treated oral cancer cells. Using oral cancer and non-malignant cells, the preferential antiproliferation effects of fucoidan on oral cancer cells were validated for the first time in the present study. 

**Figure 10:**
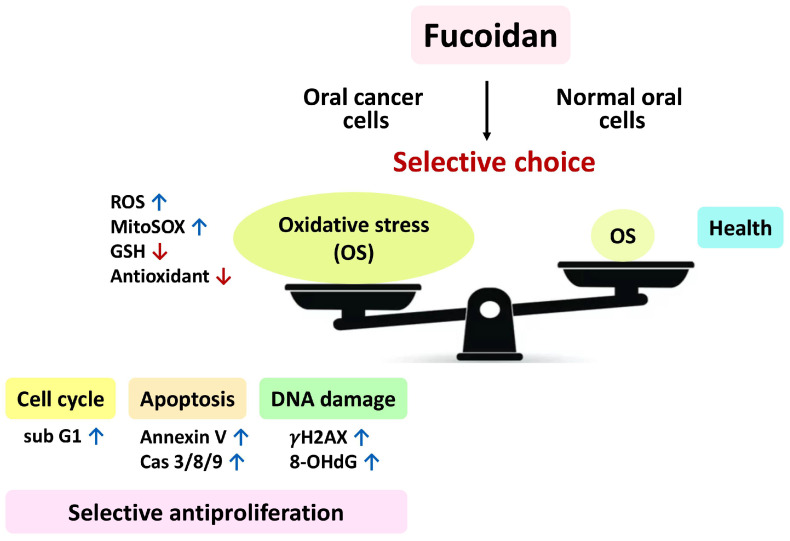
Overview of the preferential antiproliferation mechanism of fucoidan acting on oral cancer cells. In brief, fucoidan makes a preferential choice and then preferentially induces higher oxidative stress (OS) in oral cancer cells than in normal cells. In turn, this oxidative stress triggers a series of preferential responses in oral cancer cells, such as cell cycle arrest, apoptosis, and DNA damage. Finally, it causes preferential antiproliferation in oral cancer cells. In contrast, fucoidan only caused low levels of these changes, and normal cells showed healthy proliferation in the presence of fucoidan. Abbreviations: reactive oxygen species (ROS); mitochondrial superoxide (MitoSOX); glutathione (GSH); oxidative stress (OS); subG1 (subG1 phase); caspases 3/8/9 (Cas 3/8/9); 8-hydroxy-2-deoxyguanosine (8-OHdG).↓, 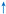, 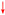 indicate making decision, upregulation, and downregulation, respectively.

Our findings demonstrate that fucoidan changed the cell cycle progression, triggered apoptosis, turned on intrinsic and extrinsic apoptosis signaling, stimulated oxidative stress, inhibited antioxidant signaling, and improved DNA damage in oral cancer cells. At the same time, non-malignant cells showed a low level of these events. These preferential changes between oral cancer and non-malignant cells contribute to the preferential antiproliferation mechanism of fucoidan (Figure 10). 

Moreover, the action of this antiproliferation mechanism was suppressed by the antioxidant NAC, indicating that fucoidan-induced antiproliferation in oral cancer cells is dependent on oxidative stress. Therefore, the contributions of the present study shed light on our understanding of preferential antiproliferation effects and the associated mechanism of fucoidan in oral cancer cells.

## Figures and Tables

**Table 1:** Primer sequences for antioxidant signaling genes.

Genes	Forward Primers (5’ → 3’)	Reverse Primers (5’ → 3’)	Accession Number
*NRF2*	GATCTGCCAACTACTCCCAGGTT	CTGTAACTCAGGAATGGATAATAGCTCC	NM_006164.5
*TXN*	GAAGCAGATCGAGAGCAAGACTG	GCTCCAGAAAATTCACCCACCT	NM_003329.4
*HMOX1*	CCTTCTTCACCTTCCCCAACAT	GGCAGAATCTTGCACTTTGTTGC	NM_002133.3
*GAPDH*	CCTCAACTACATGGTTTACATGTTCC	CAAATGAGCCCCAGCCTTCT	NM_002046.7

## Data Availability

Data are contained within the article.

## Supplementary Materials

The following supporting information can be downloaded at: https://www.mdpi.com/article/10.3390/antiox11050841/s1, Figure S1: Fucoidan drives Cas 3, 8, and 9 signaling in oral cancer cells; Figure S2: Fucoidan drives ROS and MitoSOX generation in oral cancer cells; Figure S3: Fucoidan increases the γH2AX level of oral cancer cells; Figure S4: Fucoidan increases the 8-OHdG level of oral cancer cells; Figure S5: Positive control patterns for cell cycle analysis; Figure S6: Positive control patterns for apoptosis; Figure S7: Positive control patterns for ROS analysis; Figure S8: Positive control patterns for MitoSOX analysis; Figure S9: Positive control patterns for γH2AX analysis; Figure S10: Positive control patterns for 8-OHdG analysis. The flow cytometry density plots for Figure 4, Figure 5, Figure 8 and Figure 9 are shown in Supplementary Figures S1–S4, respectively. The positive controls for cell cycle, annexin V (apoptosis), ROS, MitoSOX, γH2AX, and 8-OHdG are shown in Supplementary Figures S5–S10, respectively.
