# Supplementary material for: Brown Algae-Derived Fucoidan Exerts Oxidative Stress-Dependent Antiproliferation on Oral Cancer Cells
Source: Antioxidants (Basel). 2022 Apr 26;11(5):841. doi: 10.3390/antiox11050841 (PMC9138104; doi:10.3390/antiox11050841)
Supplement: Supplementary file 1 [file antioxidants-11-00841-s001.zip › antioxidants-1675855-SI.pdf]

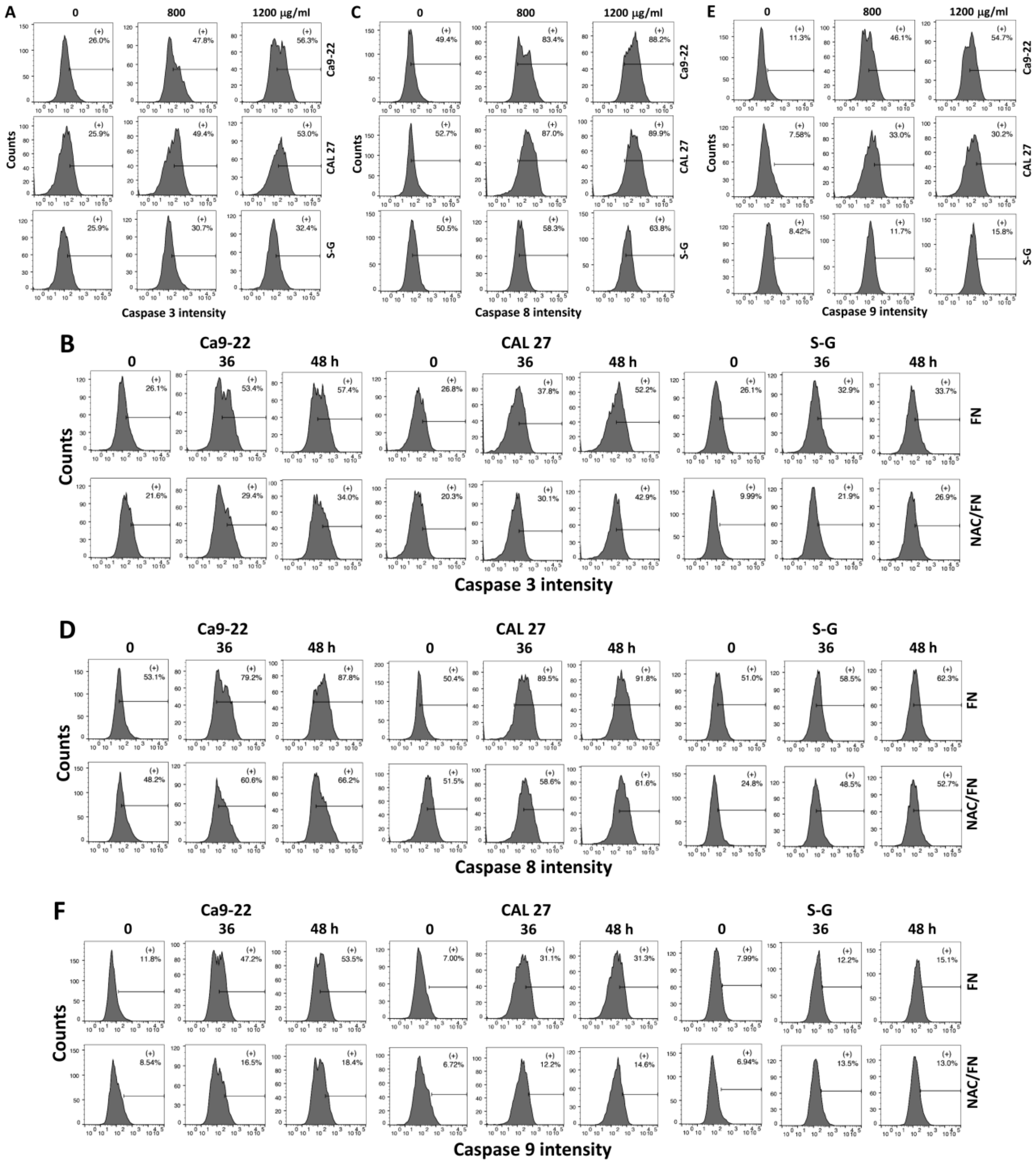

Figure S1. Fucoidan drives Cas 3, 8, and 9 signaling in oral cancer cells. (**A**, **C**, and **E**) Cas 3, 8, and 9 flow cytometry density plots. Oral cancer (Ca9-22 and CAL 27) and non-malignant cells (S-G) were treated with fucoidan (control, 800, and 1200 μg/ml) for 0 and 48 h. (+) indicates a high level of Cas 3, 8, and 9. (**B**, **D**, and **F**). The effect of oxidative stress on Cas 3, 8, 9 activations of fucoidan-incubated oral cancer cells. After NAC incubation or not, cells were treated with fucoidan (1200 μg/ml) for 0, 36, and 48 h. These are flow cytometry density plots for Figure 4. In general, the actual setting for the windows in (**A**, **C**, and **E**) is based on similar (+) (%) of the controls between different cell lines. For NAC pretreatment, the actual setting for the windows in (**B**, **D**, and **F**) is based on the same caspase intensity of the controls between different cell lines.

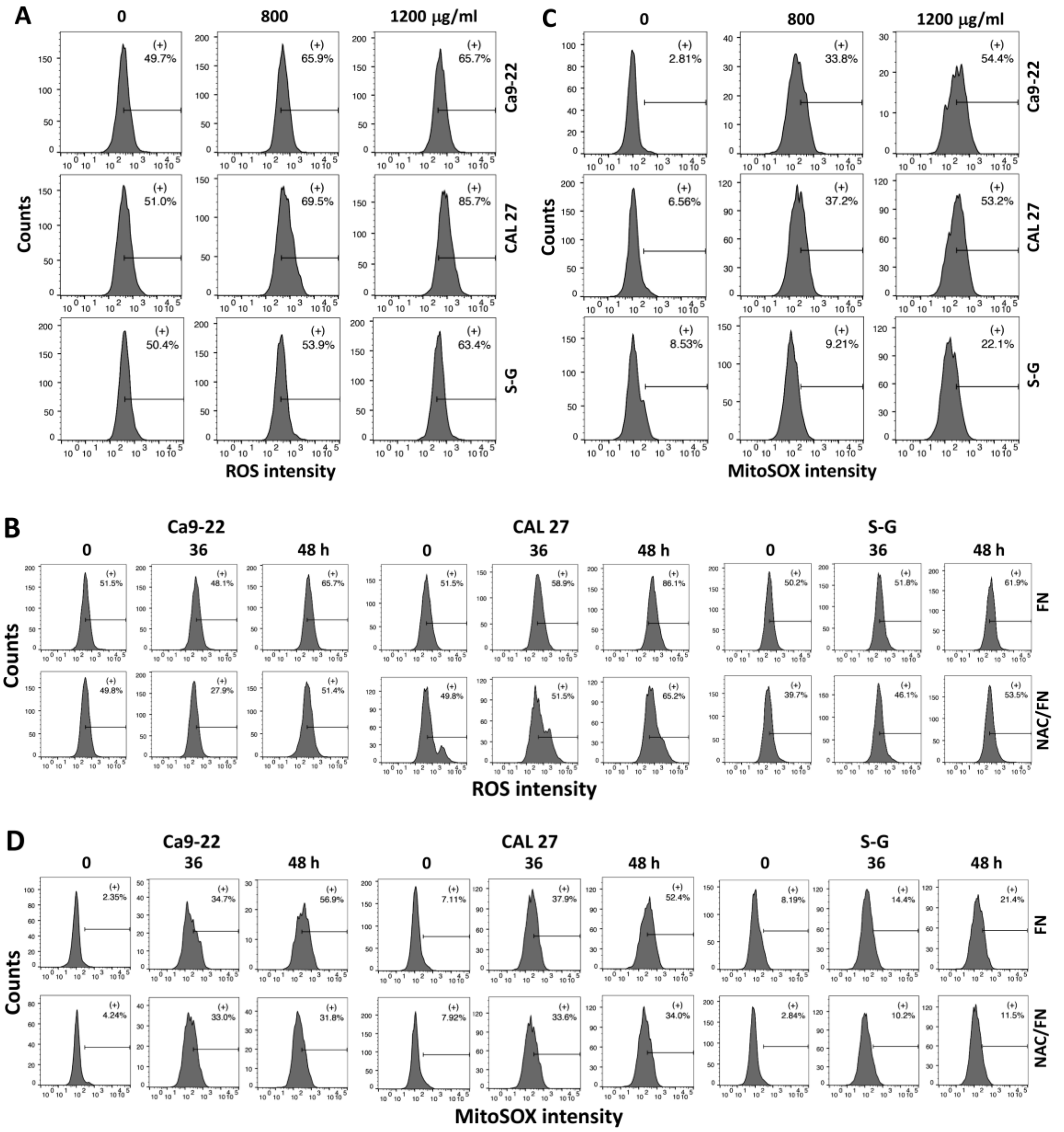

Figure S2. Fucoidan drives ROS and MitoSOX generation in oral cancer cells**.** (**A** and **C**) ROS and MitoSOX flow cytometry density plots. Oral cancer (Ca9-22 and CAL 27) and non-malignant cells (S-G) were treated with fucoidan (control, 800, and 1200 µg/ml) for 0 and 48 h. (+) indicates high levels of ROS and MitoSOX. (**B** and **D**) The effect of oxidative stress on ROS and MitoSOX generation and the GSH depletion of fucoidan-incubated oral cancer cells. After NAC incubation or not, cells were treated with fucoidan (1200 µg/ml) for 0, 36, and 48 h. These are flow cytometry density plots for Figure 5. In general, the actual setting for the windows in (**A** and **C**) is based on similar (+) (%) of the controls between different cell lines. For NAC pretreatment, the actual setting for the windows in (**B** and **D**) is based on the same ROS or MitoSOX intensity of the controls between different cell lines.

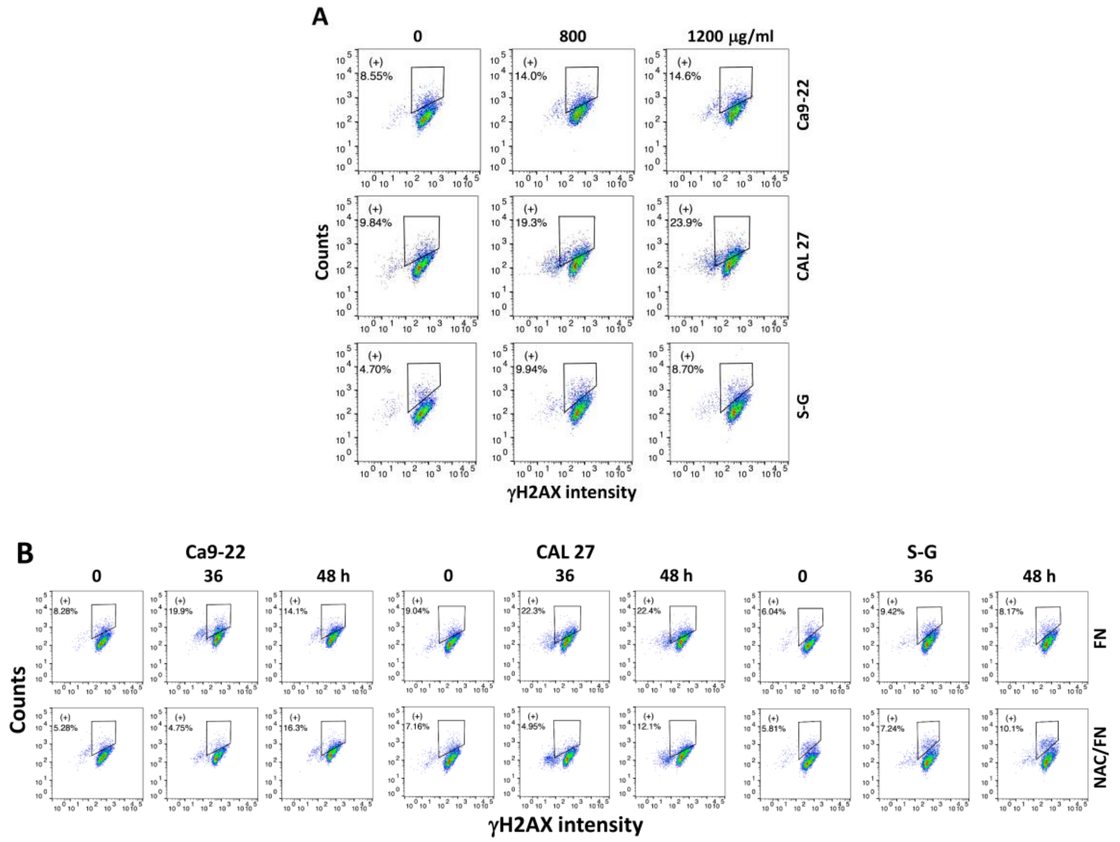

Figure S3. Fucoidan increases the γH2AX level of oral cancer cells. (**A**) γH2AX flow cytometry density plots. Oral cancer (Ca9-22 and CAL 27) and non-malignant cells (S-G) were treated with fucoidan (control, 800, and 1200 μg/ml) for 0 and 48 h. (+) indicates a high level of γH2AX. (**B**) The effect of oxidative stress on γH2AX increment in fucoidan-incubated oral cancer cells. After NAC incubation or not, cells were post-treated with fucoidan (1200 μg/ml) for 0, 36, and 48 h. These are flow cytometry density plots for Figure 8. In general, the actual setting for the windows in (**A** and **B**) is based on similar (+) (%) of the controls between different cell lines.

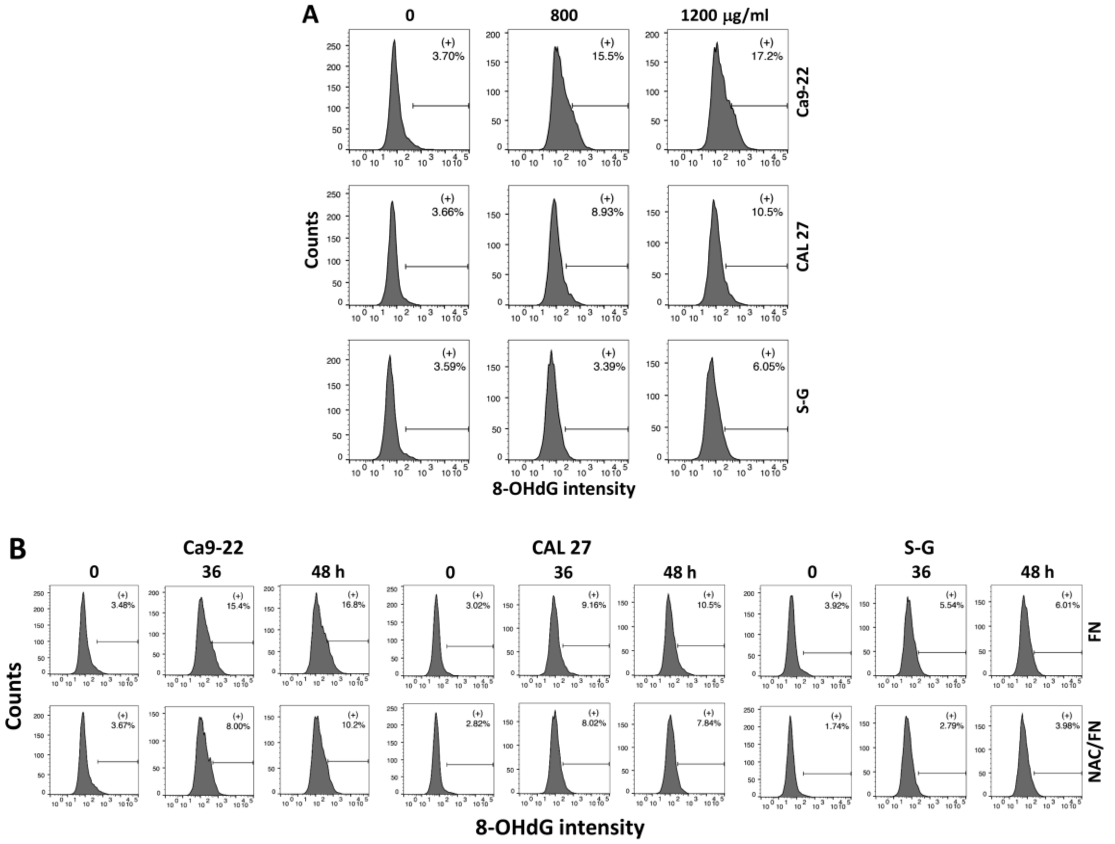

Figure S4. Fucoidan increases the 8-OHdG level of oral cancer cells. (**A**) 8-OHdG flow cytometry density plots. Oral cancer (Ca9-22 and CAL 27) and non-malignant cells (S-G) were treated with fucoidan (control, 800, and 1200 µg/ml) for 0 and 48 h. (+) indicates a high level of 8-OHdG. (**B**) The effect of oxidative stress on the 8-OHdG increment in fucoidan-incubated oral cancer cells. After NAC incubation or not, cells were treated with fucoidan (1200 µg/ml) for 0, 36, and 48 h. These are flow cytometry density plots for Figure 9. In general, the actual setting for the windows in (**A**) is based on similar (+) (%) of the controls between different cell lines. For NAC pretreatment, the actual setting for the windows in (**B**) is based on the same 8-OHdG intensity of the controls between different cell lines.

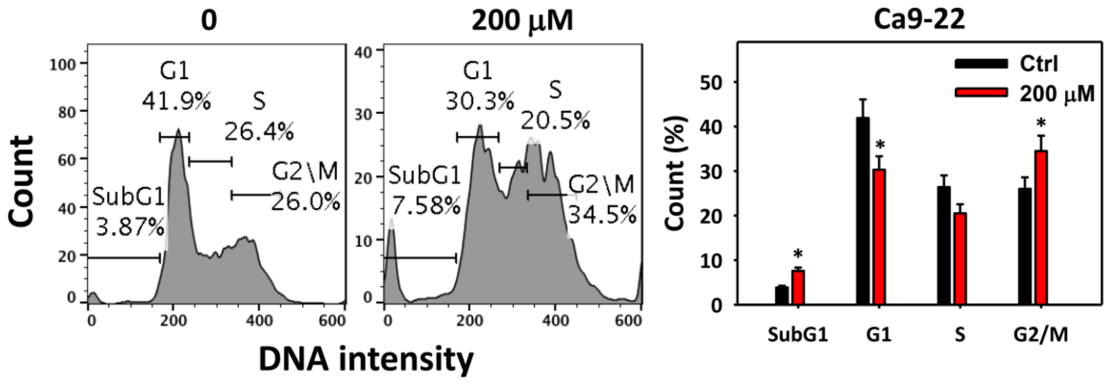

Figure S5. Positive control patterns for cell cycle analysis. Flow cytometry density plots and summary statistics were shown. Cells (Ca9-22) were treated with H_2O_2 (200 μM) for 24 h. * indicate $P < 0.05$ (*t*-test). Data = mean ± SD (n = 3).

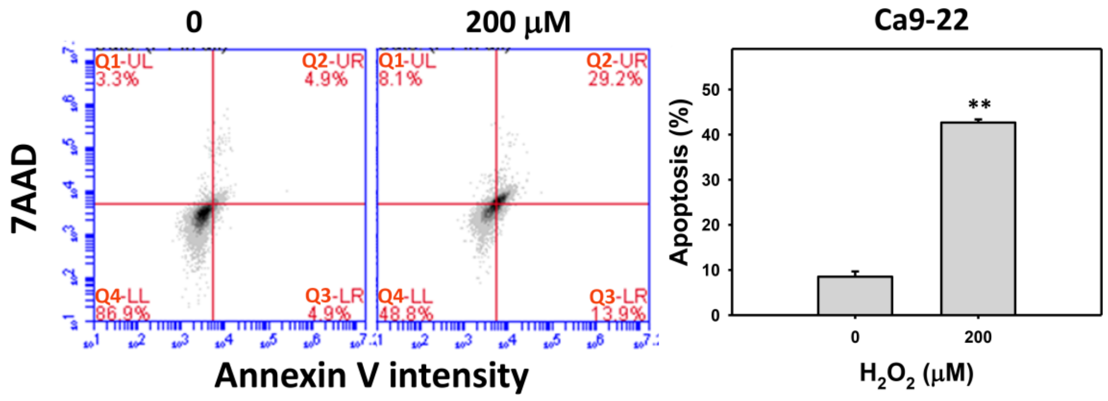

Figure S6. Positive control patterns for apoptosis. Flow cytometry density plots and summary statistics were shown. Cells (Ca9-22) were treated with H_2O_2 (200 μM) for 24 h. ** indicates $P < 0.01$ (*t*-test). Data = mean ± SD (n = 3).

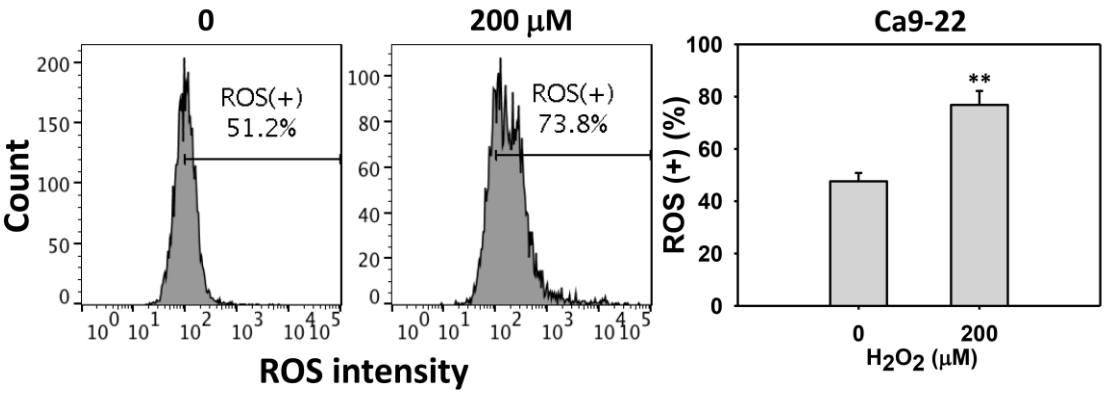

Figure S7. Positive control patterns for ROS analysis. Flow cytometry density plots and summary statistics were shown. Cells (Ca9-22) were treated with H_2O_2 (200 μM) for 24 h. ** indicates $P < 0.01$ (*t*-test). Data = mean ± SD (n = 3).

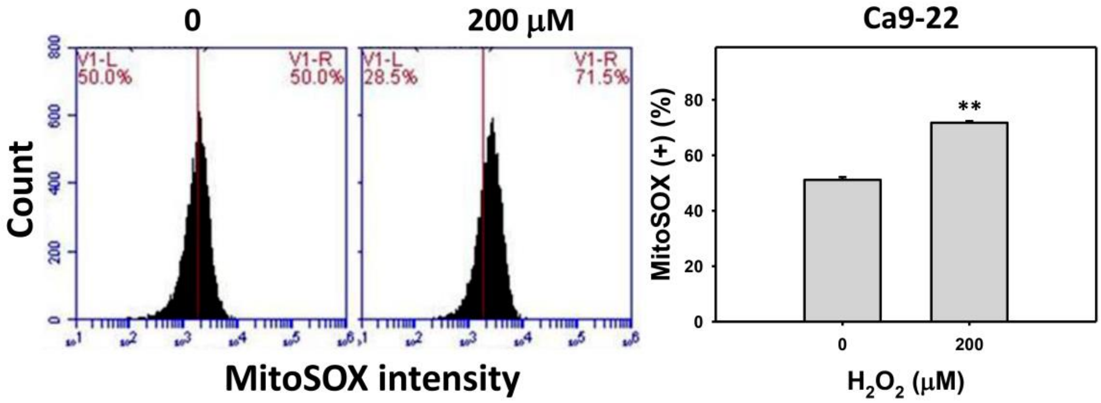

Figure S8. Positive control patterns for MitoSOX analysis. Flow cytometry density plots and summary statistics were shown. Cells (Ca9-22) were treated with H_2O_2 (200 μM) for 24 h. ** indicates $P < 0.01$ (*t*-test). Data = mean ± SD (n = 3).

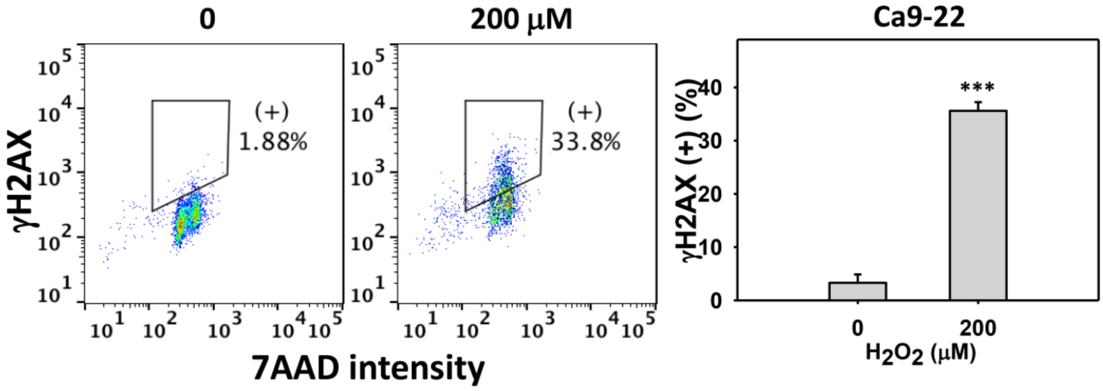

Figure S9. Positive control patterns for γH2AX analysis. Flow cytometry density plots and summary statistics were shown. Cells (Ca9-22) were treated with H_2O_2 (200 μM) for 24 h. *** indicates $P < 0.001$ (*t*-test). Data = mean ± SD (n = 3).

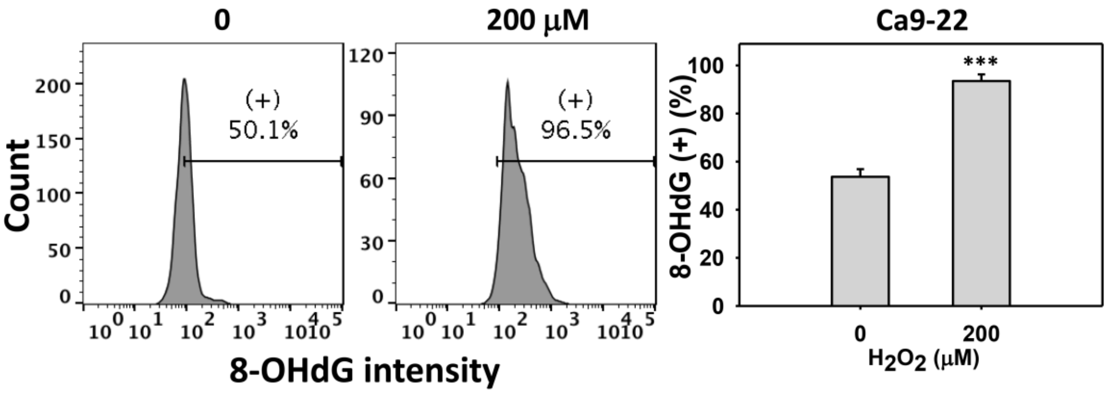

Figure S10. Positive control patterns for 8-OHdG analysis. Flow cytometry density plots and summary statistics weer shown. Cells (Ca9-22) were treated with H_2O_2 (200 μM) for 24 h. *** indicates $P < 0.01$ (*t*-test). Data = mean ± SD (n = 3).
